# Case report: VEXAS syndrome: an atypical indolent presentation as sacroiliitis with molecular response to azacitidine

**DOI:** 10.3389/fimmu.2024.1403808

**Published:** 2024-05-22

**Authors:** Roberto Pereira da Costa, Guilherme Sapinho, Matilde Bandeira, Joana Infante, Tiago Marques, Carla Mimoso Santos, João Forjaz de Lacerda, João Eurico Fonseca, José Carlos Romeu

**Affiliations:** ^1^ Rheumatology Department, Unidade Local de Saúde Santa Maria, Centro Académico de Medicina de Lisboa, Lisbon, Portugal; ^2^ Instituto de Medicina Molecular, Faculdade de Medicina, Universidade de Lisboa, Centro Académico de Medicina de Lisboa, Lisbon, Portugal; ^3^ Hematology and Marrow Transplantation Department, Unidade Local de Saúde Santa Maria, Centro Académico de Medicina de Lisboa, Lisbon, Portugal; ^4^ Infectious Diseases Department, Unidade Local de Saúde Santa Maria, Centro Académico de Medicina de Lisboa, Lisbon, Portugal

**Keywords:** VEXAS syndrome, autoinflammatory, ruxolitinib, azacitidine, case report

## Abstract

VEXAS syndrome is a recently described autoinflammatory syndrome caused by the somatic acquisition of *UBA1* mutations in myeloid precursors and is frequently associated with hematologic malignancies, chiefly myelodysplastic syndromes. Disease presentation can mimic several rheumatologic disorders, delaying the diagnosis. We describe a case of atypical presentation resembling late-onset axial spondylarthritis, later progressing to a systemic inflammatory syndrome with chondritis, cutaneous vasculitis, and transfusion-dependent anemia, requiring high doses of steroids. Ruxolitinib was used as the first steroid-sparing strategy without response. However, azacitidine showed activity in controlling both inflammation and the mutant clone. This case raises the question of whether azacitidine’s anti-inflammatory effects are dependent on or independent of clonal control. We discuss the potential relevance of molecular remission in VEXAS syndrome and highlight the importance of a multidisciplinary team for the care of such complex patients.

## Introduction

VEXAS syndrome is a novel entity that was first described in 2020 by Beck et al. (1). The acronym stands for some of its most distinctive features: V—vacuoles in erythroid and myeloid precursors cells, E—low levels of E1 (a ubiquitin-activating enzyme), X—the mutated *UBA1* gene located on the X chromosome, A—autoinflammatory syndrome, and S—somatic mutations.

This acquired autoinflammatory disease is caused by inactivating mutations of the X-linked *UBA1* gene that lead to loss of ubiquitination, subsequent accumulation of misfolded proteins, and activation of multiple inflammatory pathways. Of note, the majority of VEXAS syndrome cases that have been described are associated with a somatic mutation at p.Met41 of UBA1. However, there have been multiple reports of other mutations at the *UBA1* gene and even UBA1-negative VEXAS-like presentation of systemic autoinflammatory diseases associated with myelodysplastic syndrome (MDS) (2–6). *UBA1* variants are estimated to be present in 1/4,000 men over 50 years (7), and different mutations are responsible for a wide variety of clinical presentations and disease severity (1, 8).

VEXAS syndrome can mimic multiple inflammatory disorders, most commonly relapsing polychondritis, vasculitis, and Sweet syndrome (7). It typically presents as a progressive systemic inflammatory condition in men over 50 years of age. The most frequently described manifestations are fever, cutaneous involvement, and hematological abnormalities (9, 10). The latter can range from mild macrocytic anemia to a formal diagnosis of MDS (present in 30%–63%), monoclonal gammopathy of undetermined significance (MGUS), or myeloma (10%–25% of cases, many with concomitant MDS). However, whether the hematologic neoplasms arise from the *UBA1* mutant clone or as a consequence of clonal selection promoted by the inflammatory state remains to be clarified (11).

The lack of awareness of the medical community and the unusual constellation of symptoms and signs both contribute to the misdiagnosis and underdiagnosis of VEXAS syndrome. The treatment of this hemato-inflammatory condition usually requires high doses of corticosteroids, as well as several steroid-sparing therapies, namely, JAK inhibitors, IL-6 inhibitors, hypomethylating agents, and allogeneic hematopoietic stem cell transplant. The prognosis is not yet fully established, with reported 5-year survival rates ranging from 63% to 83% (9).

**Figure 1:**
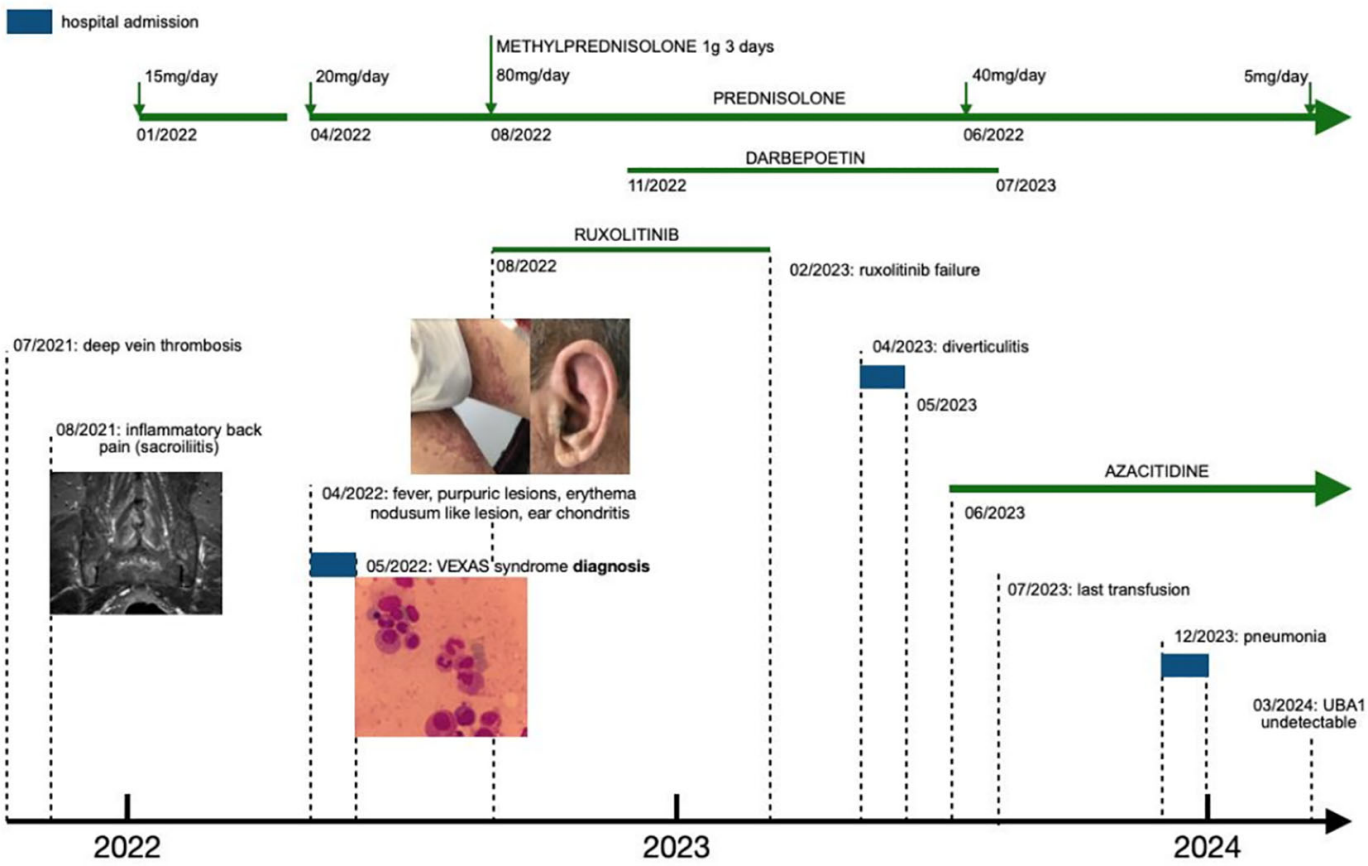
Timeline of symptoms and interventions. Refer to **Figures 2**, **3** for a more detailed view of the images.

We describe a challenging case of VEXAS syndrome with an indolent presentation as sacroiliitis, later progressing to a severe systemic inflammatory syndrome and transfusion-dependent anemia, who responded to azacitidine after prolonged steroid dependency and ruxolitinib failure (see **Figure 1**). We additionally provide evidence of molecular response to azacitidine, opening the door to residual disease monitoring by next-generation sequencing (NGS).

## Case description

A 74-year-old man of Caucasian ancestry presented to our Rheumatology Clinic in February 2022 with a 6-month history of fatigue, anorexia, nighttime lower back pain, and prolonged morning stiffness. He had a history of lower extremity deep vein thrombosis (DVT) in July 2021 but had no relevant medical history. Blood workup revealed macrocytic anemia (hemoglobin [Hb] 8.8 g/dL, mean corpuscular volume [MCV] 101.0 fL) and increased acute-phase reactants [C-reactive protein (CRP) 4.27 mg/dL, ferritin 994 ng/L, erythrocyte sedimentation rate (ESR) 113 mm/h]. There were no other cytopenias nor significant changes in renal, hepatic, or thyroid function. Hemolysis was not present and vitamin B_12_, folate, copper, and zinc levels were normal.

**Figure 2:**
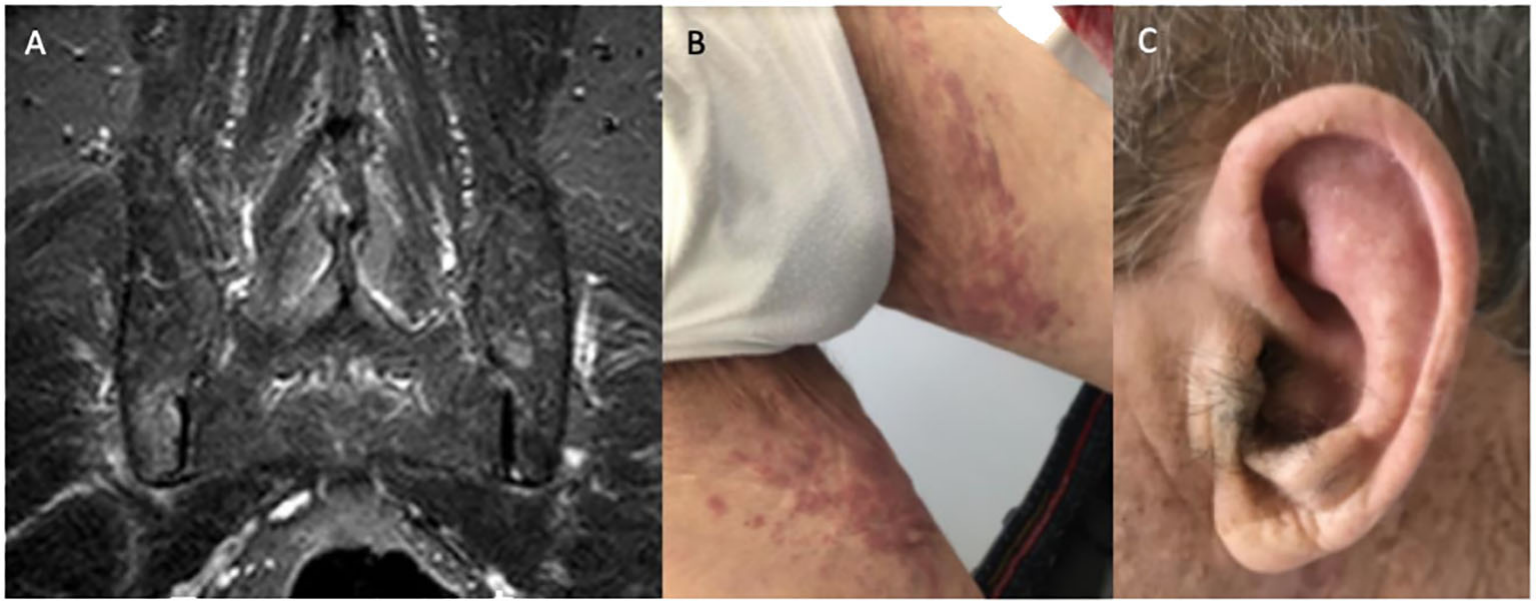
**(A)** Magnetic resonance imaging documenting active sacroiliitis; **(B)** lower limb purpuric lesions; **(C)** ear chondritis.

The patient had been prescribed prednisolone 15 mg daily for 3 weeks by his general practitioner, with partial improvement of his symptoms and laboratory tests (Hb 11.0 mg/dL, MCV 105.7 fL, CRP 0.58 mg/dL, ESR 115 mm/h). Magnetic resonance imaging (MRI) of the sacroiliac joints was obtained and showed bilateral sacroiliitis with moderate asymmetric inflammatory activity (see **Figure 2**). Additionally, a color Doppler ultrasound of the head, neck, and upper extremities and positron emission tomography (FDG-PET/CT) were performed, with no evidence of large vessel vasculitis. FDG-PET/CT was normal apart from a diffusely increased FDG uptake in the bone/bone marrow, suggestive of non-specific medullary stimulation.

After the hematology review, anemia was first labeled as anemia of chronic disease, possibly related to inflammation secondary to an undiagnosed inflammatory condition.

In April 2022, the patient was admitted to the Infectious Diseases Department due to a 10-day history of fever (1–2 spikes/day with a maximum temperature of 39.0°C) while on prednisolone 7.5 mg daily. On admission, he presented with inflammatory lower back pain, bilateral lower limb purpuric skin lesions (see **Figure 2**), worsening anemia (Hb 8.5 g/dL, MCV 100.0 fL), and raised CRP and ESR (10.5 mg/dL and 88 mm/h, respectively). Prednisolone was stopped and the patient was started on diclofenac 50–75 mg twice daily. A complete workup for fever of unknown origin was performed. Computed tomography (CT) of the neck, chest, abdomen, and pelvis and transesophageal echocardiogram were unremarkable. Blood cultures, serologic screening for infectious causes, and a comprehensive autoimmune panel were negative.

A skin biopsy was performed when the patient developed an erythematous nodular skin lesion on the dorsum of the foot with findings of non-specific neutrophilic vasculitis. Progressively worsening anemia with new transfusion dependency (minimum Hb 5.9g/dL) prompted a bone marrow aspiration and biopsy. The initial report noted a hypercellular marrow with erythroid hypoplasia, an increased myeloid to erythroid ratio (M:E 7:1), and no significant morphological changes nor left shift.

**Figure 3:**
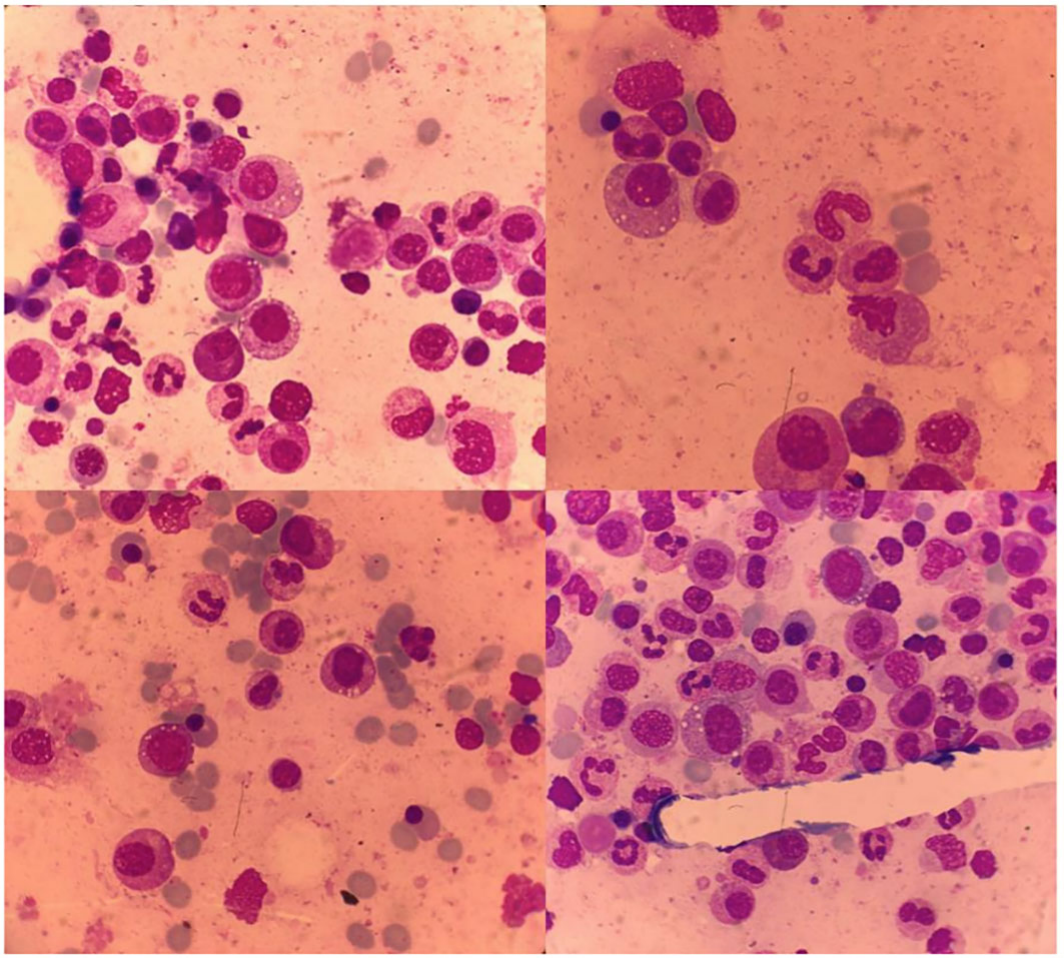
Bone marrow with vacuolization of myeloid and erythroid precursor cells.

On the third week of hospital admission with persistent fever, slightly improved inflammatory lower back pain, and persistently elevated acute-phase reactants, the patient developed auricular chondritis (see **Figure 2**). Hence, a diagnosis of relapsing polychondritis was assumed. Prednisolone 20 mg daily was started with the resolution of the fever, chondritis, and skin lesions. Due to the persistence of disproportionate macrocytic anemia after discharge, a review of the bone marrow aspirate was requested, showing extensive vacuolization of myeloid and erythroid precursors, without features of MDS (see **Figure 3**). No cytogenetic abnormalities were identified in FISH or karyotyping, and a 30-gene NGS panel was negative for the most common MDS-related gene mutations.

Therefore, a diagnosis of VEXAS syndrome was suspected and subsequently confirmed through the identification of the *UBA1* p.Met41Thr variant by NGS, with a variant allele fraction (VAF) of 71.25%.

Soon after discharge, while on prednisolone 12.5 mg daily, the patient was readmitted due to recrudescence of fever, back pain, severe anemia, and a rise in acute-phase reactants. He received intravenous methylprednisolone pulse therapy (1 g daily for 3 days) followed by prednisolone 1 mg/kg/day (80 mg) and ruxolitinib (starting at 15 mg twice daily). After an initial response, symptomatic relapse occurred upon prednisolone reduction below 30 mg. Despite titrating ruxolitinib dose up to 25 mg twice daily and starting darbepoetin alfa support (maximum 300 μg weekly), tapering below 30 mg of prednisolone was not possible.

A bone marrow aspirate was repeated and confirmed persistent vacuolization but no progression to MDS or acute myeloid leukemia (AML). Given the severe inflammatory behavior and absence of progression to MDS, a switch to tocilizumab was decided. However, before starting tocilizumab, the patient was admitted for diverticulitis with a local abscess, further increasing the risk of bowel perforation reported with this drug (12). A decision was then made to start azacitidine at the standard dose of 75 mg/day/m^2^ administered for 5 + 2 days (28-day cycles). Azacitidine was started in June 2023, allowing progressive steroid tapering, from an initial dose of 40 mg of prednisolone daily to a minimum dose of 5 mg daily as of March 2024. No significant adverse events were noticed. The patient has received 9 cycles of azacitidine and is currently in clinical remission, with CRP <0.5 mg/dL, ESR <50 mm/h, and Hb >12g/dL, and has been transfusion-free for 8 months. Importantly, after 9 months of therapy, bone marrow reassessment was consistent with a significant decrease in precursor cell vacuolization with no additional signs of dysplasia, and the *UBA1* p.Met41Thr VAF decreased from 71.25% to being undetectable, suggesting a deep molecular response.

During the entire follow-up, the existence of a multidisciplinary team allowed for the assessment of different comorbidities. The patient received prophylaxis with acyclovir 200 mg twice daily and trimethoprim–sulfamethoxazole 960 mg thrice weekly while on prednisolone >20 mg/day. Immunizations against SARS-CoV-2, influenza, *Streptococcus pneumoniae*, and herpes zoster were also administered. Despite this, the patient experienced several infectious complications, including a dental abscess, two viral upper respiratory tract infections (one caused by respiratory syncytial virus), an oral candidiasis, and two admissions for acute diverticulitis and severe bilateral pneumonia requiring high-flow oxygen therapy (presumably bacterial). He also suffered two osteoporotic vertebral fractures, despite early institution of antiresorptive treatment with zoledronic acid and calcium carbonate and cholecalciferol supplementation. Given the history of DVT and the thrombotic risk associated with VEXAS syndrome, secondary thromboprophylaxis with rivaroxaban was kept during the entire treatment course.

## Patient perspective

Throughout the whole process of the disease, the patient was supported by his wife and family. The novelty of the disease that contributed to a delayed diagnosis affected both the patient and his caregivers. A joint statement by the patient and his wife (main caregiver) was collected: “*The initial phase of the disease was very complicated as the symptoms were so pronounced that they prevented my husband from having an active lifestyle as before. This disease has had an enormous impact on the autonomy of my husband. That and the high number of hospital visits limited the activities we had planned for our retirement years. We felt that the final diagnosis was only possible with the dedication and collaboration of a multidisciplinary team. Getting to know the disease’s name and what limited information there is about it was essential to start to understand what was happening. We are grateful to all the staff and for having the chance to do this treatment, that allowed us to regain some independence.*”

## Discussion

The case presented here illustrates the difficulty in diagnosing and managing VEXAS syndrome. Indolent symptom onset with mild non-specific findings can be misleading. We present a difficult-to-diagnose case of a 74-year-old man with symptoms spanning a 9-month period before diagnosis. The first manifestation was likely the unprovoked DVT. Patients with VEXAS syndrome are at a high risk of venous thromboembolic events, with a reported prevalence of 41% and a recurrence rate of 41% (13). Obiorah et al. have reported two episodes of deep vein thrombosis as an initial manifestation in a cohort of 16 VEXAS syndrome patients (14). Of interest, Khider et al. demonstrated that systematically screening for the UBA1 mutation among 97 male patients aged over 50 years after a first thromboembolic event did not yield discernible benefit. This sheds light on the limited utility of such screening in this specific cohort, highlighting the overall high prevalence of thromboembolic events in older patients without an underlying autoinflammatory disease (15).

The DVT was followed by inflammatory lower back pain with evidence of sacroiliitis on MRI over half a year later. At this time, the clinical presentation resembled late-onset axial spondylarthritis; however, the marked inflammatory syndrome and macrocytic anemia remained to be clarified. In fact, many of the described VEXAS syndrome cases in the literature were first classified as an inflammatory rheumatic disease (7). Initial symptoms suggesting an axial spondylarthritis are quite atypical. Magnol et al. described a case of a patient with axial and peripheral spondylarthritis who, after a sustained clinical response over 2 years under TNF inhibitor therapy, developed anterior uveitis, ear and nose chondritis, neutrophilic dermatosis, medium vessel vasculitis, and macrocytic anemia. After the first description of VEXAS syndrome, the authors revisited the case and were able to confirm the molecular diagnosis (16).

In the presented case, the development of fever, severe anemia, skin findings, chondritis, and a marked inflammatory syndrome widened the differential diagnosis. At this point, symptoms seemed to be consistent with steroid-dependent relapsing polychondritis, but the transfusion-dependent macrocytic anemia was yet to be explained. Only a second review of the marrow aspirate identified vacuolization of erythroid and myeloid precursors as a prominent feature. These previously overlooked marrow changes were very important clues to the final diagnosis, highlighting the importance for hematopathologists to be familiar with this entity and the need for physicians across various specialties to communicate and collaborate throughout the diagnostic process.

After overcoming the diagnostic challenge, physicians had to face another challenge: treatment. While awaiting the results of the first clinical trials dedicated to VEXAS syndrome, the management of these patients has been informed by case reports and case series. A significant part of the treatment options discussed in the literature are chosen based on the symptoms of the patient and the rheumatological/hematological disease that most closely aligns with their condition. A rational approach has been targeting the dominant phenotype of the disease, either 1) using MDS-directed therapies such as azacitidine, lenalidomide, or allogeneic stem cell transplant for those with features consistent with a hematologic neoplasm or 2) targeting inflammatory hyperactivation, both unspecifically (steroids, JAK inhibitors, cyclosporine) or by blocking specific cytokines (anti-IL1, anti-IL6, anti-TNF) in those whose clinical behavior resembles classical autoinflammatory diseases (17).

This line of thought was followed to select the first line of treatment for this patient. In the absence of an overt hematologic neoplasm, ruxolitinib was chosen as frontline therapy based on the results published by Heiblig et al. suggesting the superiority of ruxolitinib over other JAK inhibitors, as well as higher response rates when compared with tocilizumab (18).

After ruxolitinib failure, taking into account the predominantly inflammatory behavior, the absence of dysplasia or increased marrow blasts, the high number of hospital visits required for azacitidine treatment, and azacitidine’s higher myelotoxicity, tocilizumab was chosen as second-line therapy. However, upon the moment of treatment decision, the patient had severe diverticulitis, thus increasing the already known risk of tocilizumab for bowel perforation. This prompted the switch to azacitidine, which was associated with a remarkable clinical recovery and sustained transfusion independence, allowing for steroid tapering to low doses. The control of systemic inflammation with azacitidine raises the question of whether azacitidine has direct anti-inflammatory properties in VEXAS syndrome patients or whether reducing the clonal burden in the marrow is the primary driver of inflammation control. Indeed, there is evidence suggesting that azacitidine can modulate the number and function of regulatory T cells in MDS, as well as clinical evidence of immunomodulatory effects (19). Perhaps this strategy circumvents the need to determine the “dominant phenotype” by acting both on inflammation and on the clonal component.

In this case, we demonstrated both success in controlling inflammation and in attaining clonal suppression, as seen by a significant decrease in *UBA1* VAF in marrow samples over time. Sockel et al. presented two cases of molecular response to azacitidine in newly diagnosed VEXAS syndrome patients (20). Raajimakers et al. have described three cases of retrospectively diagnosed VEXAS syndrome. In their report, two patients, after failed attempts of treatment with azathioprine, mycophenolic acid, intravenous immunoglobulin, and anakinra, had a complete clinical response and almost eradication of the mutated clone while on treatment with azacitidine. One of these patients has been with no disease-directed treatment for 4.5 years with persistent remission and suppression of the mutant UBA1 clone (21). We hereby present a case of molecular response to azacitidine after ruxolitinib failure, confirming the potential for disease modification with azacitidine in VEXAS syndrome, even in a refractory setting. Nonetheless, a better understanding of the precise mechanisms of inflammation and clonal dynamics in VEXAS and of azacitidine’s multiple modes of action should be given priority for better management of the disease.

As a much less genetically heterogeneous entity than MDS or AML, longitudinal monitoring of *UBA1* burden in VEXAS patients may have a role in guiding therapeutic decisions. Attainment of molecular responses (especially if sustained over time) may open the door to discuss time-limited therapy and treatment-free intervals in selected cases. However, these observations need to be replicated in prospective multicentric cohorts to validate the prognostic value of molecular clearance and define the thresholds of clinical significance. Until further data emerge, it is unclear whether stopping azacitidine will be possible in our patient.

Lastly, we emphasize that the management of VEXAS syndrome requires a dedicated multidisciplinary team and a special focus on supportive care. We believe that most adverse events (including numerous infections) experienced by this patient were attributable to prolonged steroid exposure. Therefore, steroid-sparing strategies to mitigate these risks should be a research priority.

## Data availability statement

The original contributions presented in the study are included in the article/supplementary material. Further inquiries can be directed to the corresponding author.

## Ethics statement

Written informed consent was obtained from the individual(s) for the publication of any potentially identifiable images or data included in this article. Written informed consent was obtained from the participant/patient(s) for the publication of this case report.

## Author contributions

RP: Writing – original draft, Writing – review & editing, Conceptualization, Data curation, Investigation, Methodology. GS: Conceptualization, Data curation, Investigation, Methodology, Writing – original draft, Writing – review & editing. MB: Conceptualization, Data curation, Investigation, Methodology, Writing – original draft, Writing – review & editing. JI: Conceptualization, Investigation, Writing – review & editing. TM: Conceptualization, Investigation, Writing – review & editing. CS: Conceptualization, Investigation, Writing – review & editing. JL: Conceptualization, Supervision, Writing – review & editing. JF: Conceptualization, Supervision, Writing – review & editing. JR: Conceptualization, Investigation, Supervision, Writing – review & editing.
